# 'Do Fever-Sugar-Swallow Protocols improve nurses' and physicians' satisfaction with the management of fever, hyperglycemia and dysphagia in stroke patients: A pre-and post-implementation survey of the “Quality in Acute Stroke Care” (QASC) Program‘

**DOI:** 10.1016/j.ijnsa.2025.100374

**Published:** 2025-06-30

**Authors:** Anne-Kathrin Cassier-Woidasky, Winfried Zinn, Sandy Middleton, Simeon Dale, Waltraud Pfeilschifter

**Affiliations:** a Saarland University of Applied Sciences, Saarbruecken, Germany; b Trier University, Department of Nursing Sciences, Trier, Germany; c PatE-Stiftungs-UG, Bermuthshain, Germany; d Nursing Research Institute, St Vincent’s Health Network Sydney and Australian Catholic University, Sydney, Australia; e School of Nursing, Midwifery and Paramedicine, Australian Catholic University, Sydney, Australia; f Klinikum Lueneburg, Department of Neurology and Clinical Neurophysiology, Lueneburg, Germany

**Keywords:** Fever and Hyperglycemia and dysphagia management, Job satisfaction, Nurse led stroke care, Nurse’s role, Professional autonomy, Self efficacy

## Abstract

**Background:**

Nurses play a key role for delivery of high-quality patient care in the interprofessional stroke team. Nurses’ autonomy and advanced competencies are essential to a positive work environment, yet, are not well developed in Germany.

**Objective:**

To assess whether using the Fever-Sugar-Swallow (FeSS) Protocols in stroke care improves: a) nurses’ satisfaction regarding their ability to autonomously manage fever, hyperglycemia, and dysphagia; and b) physicians’ satisfaction regarding the management of these parameters in German stroke units.

**Design:**

A pre-test/post-test survey administered to nursing and medical staff in eight stroke units was conducted prior to and following implementation of the FeSS Protocols.

**Methods:**

An online survey, comprising items regarding global job satisfaction, the target parameters and control parameters, was distributed to participating stroke units. T-tests were calculated for significance between pre-post implementation, Cohen’s d was used to measure the effect size.

**Results:**

A total of 308 respondents (nurses *n* = 112 [pre], *n* = 65 [post]; physicians *n* = 82 [pre], *n* = 49 [post]) from 8 stroke units completed the survey (response rates: 49 % nurses; 53 % physicians). There were no significant differences regarding global job satisfaction, but nurses were significantly more likely to report improvements in autonomy regarding fever management (able to act autonomously (*p* = 0.006) and competently (*p* < 0.001)); perceived self-efficacy (*p* < 0.001), regarding blood glucose management (able to act timely (*p* = 0.002) and competently (*p* = 0.007); perceived self-efficacy (*p* < 0.001)), and dysphagia management (able to act autonomously (*p* = 0.005) and timely (*p* = 0.007)). In the medical profession, improvements were reported in training activities of new colleagues, not only regarding FeSS parameters fever (*p* < 0.001) and dysphagia (*p* < 0.001), but also regarding management of other clinical issues which were not the subject of our intervention (restlessness (*p* < 0.001), and sleep disorders (*p* < 0.001)).

**Conclusions:**

Despite a tightly-regulated mandatory quality assurance system with well-established therapy guidelines, the implementation of the FeSS Protocols in German stroke units revealed potential for improvement by improving autonomy for nurses as a regular part of nurses’ work clinical practice, as it is common in other countries of the world. This would not only improve the quality of care, but could also contribute to increase attractiveness of work at the stroke unit.

**Registration:**

Not registered.

**What is already known**

• Nurse initiated protocols have the power to improve quality of stroke care.
• The remuneration system does not require nurses’ independent contribution.
• The impact of nurse-initiated protocols on job satisfaction is unknown.

Alt-text: Unlabelled box

**What this paper adds**

• The Fever-Sugar-Swallow Protocol has the power to improve nurses’ self-efficacy in Germany.
• Documentation requirements alone do not foster quality of stroke care.
• The Fever-Sugar-Swallow Protocol implementation results support the relevance of multifaceted implementation processes for staff satisfaction.
• Enhanced self-efficacy and autonomy could increase attractiveness of stroke nursing.

Alt-text: Unlabelled box

## 1 Background

Nurses play a key role in providing high quality patient care in the interprofessional stroke team (Langhorne and Ramachandra, 2020). Previous research has revealed that with higher level of education of nurses, patient outcomes improve in terms of decreased mortality (Aiken et al., 2014). Focusing on stroke unit care, Middleton et al., 2011, MIDDLETON et al., 2017b have shown that supported implementation of nurse-initiated protocols to improve the management of stroke complications of fever, hyperglycemia, and dysphagia (Fever-Sugar-Swallow/ FeSS Protocols) lead to significantly improved nursing processes of stroke care and improved patient outcomes. Importantly, implementation of these protocols is more successful in stroke units where nurses report higher autonomy (Middleton et al., 2017a). Cornerstones of evidence-based stroke care are nurses’ responsibility to autonomously manage fever (temperature ≥ 37.5°), hyperglycemia (Blood Glucose Level [BGL]>180 mg/dl [10 mmol/L]), and dysphagia screening within defined limits based on a standard operating procedure (SOP) (Middleton et al., 2011). The FeSS Protocols have become part of the clinical guidelines in Australia and other countries (informme.org.au).

The Quality of Acute Stroke Care (QASC) Europe Study which supported implementation of the FeSS Protocols more broadly across Europe was carried out at 64 hospitals in 17 European countries (Middleton et al., 2023). In Germany, we initially believed that in our stroke units potential improvements would be minimal, due to well-established stroke therapy guidelines (Ringleb et al., 2021) and strict remuneration policies requiring meticulous measures and documentation. However, stroke treatment processes in Germany at the time required nurses to check with the physician prior to administering antipyretics, insulin or performing swallow screen which causes avoidable delays in therapy. Nurses’ autonomy and advanced competencies as essential parts of a positive work environment (Labrague et al., 2019) require a sound scientifically based education. Research has shown that perceptions of competence as well as opportunities for advancement are crucial for staff satisfaction and nurse retention (Aiken et al., 2013). This is not well developed in Germany (Wissenschaftsrat, 2023), but often discussed as reason for nurses’ intention to leave in the context of staff shortage (Zander et al., 2013a; Koy et al., 2015), and quality of care and staffing (Brücher and Deufert, 2019), related to nurses’ education. In Germany, as nurses’ education is regularly based on Level 4 in the European Qualification Framework Series, most nurses hold a vocational education degree with low professional autonomy. In 2023, official statistics count 2755 nurses with academic education and university degrees on Level 6 or higher, which is 0.7% out of all hospital-based nurses in Germany (Statistisches Bundesamt, 2023).

**Fig. 1:**
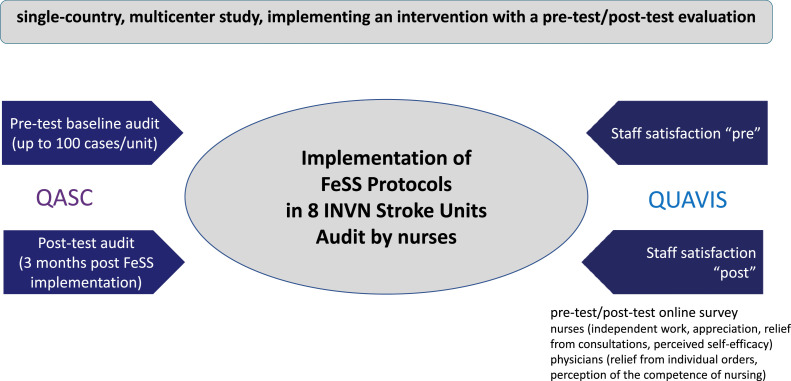
Design of the entire QASC Germany project. QASC: Quality in Acute Stroke Care; QUAVIS: Qualitätsverbesserung und Steigerung der Mitarbeiterzufriedenheit in der Pflege durch Autonomie in der Vitalparametersteuerung bei Schlaganfallpatienten auf der Stroke Unit (Improving quality and increasing staff satisfaction and autonomy in vital parameter control for stroke patients on the Stroke Unit); FeSS: Fever-Sugar-Swallow; INVN: Interdisciplinary Neurovascular Network.

We aimed to investigate whether increased professional autonomy would lead to improved staff satisfaction. We subsequently decided to implement the FeSS Protocols in eight German stroke units (QASC Germany) (Cassier-Woidasky et al., 2024). These stroke units are part of an interdisciplinary neurovascular network (INVN) across Germany. We used the same methods as described in the QASC Europe Study (Middleton et al., 2023), but adapted to local conditions (Beyer et al., 2022) (Fig. 1).

The intervention has been previously described in detail (Middleton et al., 2023), and the clinician behavior change results are published separately (Cassier-Woidasky et al., 2024). In brief the FeSS Protocols describe clear guidelines about what trained stroke nurses are required to do, if a patient with stroke suffers from elevated temperature (≥37.5°C) and blood glucose levels (>200 mg/dl) within the first 72 hours in the stroke unit, as well as how to screen for dysphagia (Middleton et al., 2023; Middleton et al., 2011). They were implemented using evidence-based implementation strategy consisting of: educational package (Forsetlund et al., 2009), audit and feedback reports (Ivers et al., 2012), local clinical champions (Flodgren et al., 2019), barrier and enabler assessments (Grimshaw et al., 2012; Cheung et al., 2012), action plans (Powell et al., 2015), reminders (Cheung et al., 2012) and external remote facilitation (Bidassie et al., 2015).

The aim of this study was to assess whether using the FeSS Protocols would improve (a) nurses’ satisfaction regarding their ability to autonomously manage fever, hyperglycemia, and dysphagia; and (b) physicians’ satisfaction regarding the management of these parameters in stroke patients before and after implementing the FeSS Protocols in German stroke units.

## 2 Methods

### 2.1 Design

We conducted a survey pre and post implementation of the FeSS Protocols with data collected from nursing and medical staff in participating stroke units. Eligible stroke units were those certified according to German Stroke Society requirements (Neumann-Haefelin et al., 2021), and members of an interdisciplinary neurovascular network (INVN), invited by the stroke network coordinator. Nurses and physicians were eligible to participate if they were involved in delivering direct patient care at the participating eight stroke units and used the protocols in any phase of the project. Our study is reported using the CROSS checklist (Sharma et al., 2021).

### 2.2 Instrument

We conducted a literature search to identify appropriate questionnaires that could be used to investigate staff satisfaction and perceptions focusing on stroke care. No suitable German-language instrument could be identified and stroke-specific related questions had not yet been addressed. Hence, we developed a questionnaire, based on and as an extension of the Metrik staff satisfaction questionnaire (Daumenlang and Zinn, 2000; Zinn and Schena, 2002; Zinn, 2004; Zinn, 2010).

The instrument used in this study consisted of two parts. The first part was a long-established questionnaire that covers various topics within the healthcare system. This section served as a quasi-control group, as it included comparative data from over 128 surveys conducted in recent years within the hospital setting (www.metrik.de/befragung/referenz.html). The second part was a newly developed questionnaire designed to assess the Fever-Sugar-Swallow Protocol implementation. This section aimed to evaluate whether the implementation focussing on how to use the FeSS Protocols would have an impact.

To obtain insights into stroke care, and to develop stroke related questions, the statistician conducted open explorative interviews with stroke unit staff members. He involved all three professional groups - nurses, physicians, and speech therapists - and brought them together in a working group. The aim was to explore which thematic areas and specific questions were relevant to the project. This consisted of several rounds of feedback and consensus-building among the researchers; the nurses, therapists and doctors which gave the interviews, and external experts, which are familiar to stroke care in Germany. A questionnaire draft was created based on these interviews. This draft focused on the perspective of the doctors and nurses, reflecting

• satisfaction of nursing staff in terms of ability to act independently, appreciation and perceived self-efficacy and
• satisfaction of doctors in terms of relief from the writing of individual patient orders (as opposed to having standard operating procedures) and perception of nurses’ autonomy and competency to manage the FeSS parameters.

Speech therapists were involved in developing the parts of the questionnaire that required the management of dysphagia by nurses and physicians in stroke cares. After reviewing and refining by the German members of the project team, the draft was checked by experienced research staff for comprehensibility and feasibility and by other stroke experts for accuracy of content. The draft underwent a pre-test by independent external testers representing the target group. The new questionnaire was pre-tested with a small group of 17 participants who completed the survey and provided handwritten comments on any unclear sections or suggestions for improvements. Following this, the optimized questionnaire was used in both the pre- and post-tests, without further changes. The analysis focused on individual items; psychometric evaluation has not been conducted. Questions were tested for face validity by experienced stroke nurses, physicians, and speech therapists. In total, the questionnaire contained 164 items and comprised three parts:

(1) Global job satisfaction with 35 questions regarding general topics of work environment and seven statements on interprofessional collaboration as well as 13 statements regarding individual aspects of job satisfaction, to be rated on a five-point Likert scale. A sample item was “I am happy with my working time arrangements.”
(2) Symptoms focusing the three target parameters (how to manage **fe**ver, hyperglycemia (**s**ugar), **s**wallowing: FeSS) with 13 statements each. Following the same scheme, we added four other symptoms as a control. We used symptoms that are common problems in stroke care but were not part of the protocol (how to manage blood pressure (**B**lutdruck), restlessness (**U**nruhe), pain (**S**chmerz), sleep disorders (**S**chlafstörungen): BUSS).
  Nurses and physicians were asked to rate competence, autonomy, cooperation, perceived relevance of their contribution, and perceived specialized training activities regarding these parameters in a five-point Likert scaled format (1 completely true, 2 largely true, 3 somewhat true, 4 somewhat not true, 5 not true at all). A sample item was “If a patient with stroke has elevated blood glucose levels (fever/ blood pressure….), I can act competently as a nurse/ nurses are able to act competently” Each parameter was organized as displayed in Table 1.

**Table 1:** Symptom related statements (example).

Nurses	Physicians
If a patient with stroke has elevated Blood Glucose Level (BGL)…	If a patient with stroke has elevated BGL…
…I know exactly what needs to be done.	…I know exactly what needs to be done.
…I can always react promptly.	…I can always react promptly.
…I can act independently	…I can act independently
…I feel competent in working with the doctors	…I feel competent in working with the nursing staff
…I regularly have to ask the doctors for content-related reasons.	…I regularly discuss the findings with the nursing staff
…I regularly have to ask for an order, even though I know what to do	…I only receive queries from the nursing staff when this is necessary.
…cooperation with the medical staff is very well organized.	…cooperation with the nursing staff is very well organized.
…I know when I need to call in other persons for support if necessary.	…I know when I need to call in other persons for support if necessary.
…always optimal care for the patient is guaranteed.	…always optimal care for the patient is guaranteed.
As nurses, we make a very important contribution to blood glucose management in the stroke unit.	On blood glucose management in the stroke unit, nurses make a very important contribution.
When it comes to blood glucose management in the stroke unit, the contribution of nursing care is perceived as important by the doctors.	In case of hyperglycemia in the stroke unit, I feel the nursing staff as competent
The process in case of elevated BGL is outstanding	The process in case of elevated BGL is outstanding
Regarding hyperglycemia in case of stroke, employees / colleagues are specifically trained.	Regarding hyperglycemia in case of stroke, employees / colleagues are specifically trained.
(3) We also collected demographic information: sex, highest qualification, length of employment and management position.
(4) In the post-implementation survey, we added one field for free comments. (“What do you take away from the project?”)

## 3 Data collection

**Fig. 2:**
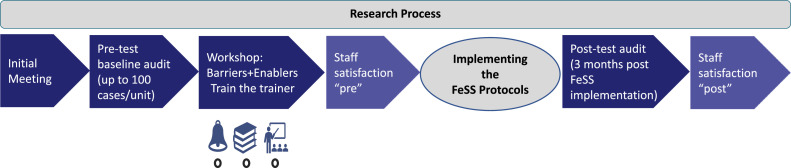
Timeline of the research process.

Each eligible staff member received a sealed letter through the site champions (staff members who were responsible for local hospital oversight of implementation of the FeSS Protocols) with a link to access the online survey and an individual transaction access number (TAN), to ensure that no multiple responses could be made. There was no link between TAN and an individuum or hospital. This procedure followed established tools by Forschungsgruppe Metrik. As we could track the number of completed surveys per hospital, we sent reminders by e-mail weekly to site champions after starting the survey, and several project newsletters (in total nine) to keep the stroke units informed on the project and to support the site champions to increase the response rate by reminding their staff to participate. Additionally, we made numerous phone calls to keep in contact with the site champions aiming to boost survey response rates. The pre-implementation survey was conducted from December 2020 until March 2021. The post-implementation survey was conducted from May 2022 until July 2022, repeating this procedure and following the same principles as before within the QASC Germany project (Cassier-Woidasky et al., 2024) (Fig. 2). The administration of the questionnaire was embedded into this process:

• Initial meeting, nomination of “site champions” by each site (responsible for local hospital oversight of implementation of the FeSS Protocols)
• Introduction to data collection to site champions
• Consenting the FeSS Protocol as standard operating procedure (SOP)
• Pre-implementation data collection for consecutive acute stroke patients, admitted up to December 31st 2019.
• Central workshops to identify barriers and enablers to use FeSS Protocols
• Train-the-trainer workshops for site champions
• **Pre-implementation staff satisfaction survey**
• Local workshops at each hospital led by site champions to implement the FeSS Protocols
• Onsite implementation training of the entire stroke unit staff at all hospitals by site champions to use the FeSS Protocols
• GO LIVE: Using the FeSS Protocols in daily clinical practice
• Onsite visits of all participating hospitals by principal investigator
• Post–implementation patient data entry (identical procedures as in pre-implementation data collection). Post-implementation data were collected from 1 September 2021, until the target sample size was reached.
• **Post implementation staff satisfaction survey**

### 3.1 Statistical analysis

Descriptive statistics such as mean, standard deviation (SD) and percentage were used to quantify data. Unpaired T-tests for independent samples were calculated to check for significance between different groups (pre-post implementation). Scores were measured using a five-point Likert scale (1 completely true to 5 not true at all) and then a mean calculated.

For statistical rigor, level of significance (alpha) was set at 0.01. Cohen’s d was used to measure the effect size as small (*d* = 0.2), medium (*d* = 0.5), and large (*d* = 0.8), as recommended by Cohen (cited from Thompson, 2007). Analyses were carried out using the software package R, missing data are excluded at the variable level. We report findings that are statistically highly significant (*p* < 0.01), according to the research questions and the target parameters.

Free text comments were grouped as per Mayring and Fenzl (2019) and following the coding principles of qualitative research (Strauss and Corbin, 1996). Respondents’ experiences became apparent, enabling comparing and contrasting. These categories emerged from the central themes provided in the comments section of the questionnaire. Two researchers, familiar with the local professional background, conducted this category building separately. They then discussed the results to merge and to reflect the appropriateness of categories based on their experience in the field.

### 3.2 Ethics approval

The QASC Germany project has been approved by the Australian Catholic University Human Research Ethics Committee (2017–114HH). QASC Germany and QUAVIS were also approved by the Human Research ethics committee of Frankfurt University Hospital on behalf of Landesaerztekammer Hessen (19–316). All data protection officers gave their consent. This online survey collected data without any inference to individuals, data entry was conducted anonymously in accordance to data protection legislation. Participation was voluntarily; consent was implied by opening and completing the questionnaire.

## 4 Results

### 4.1 Characteristics of the respondents

Staff from eight stroke units participated in the pre-implementation survey. One of them was located in a university hospital, the other ones were part of general hospitals. One stroke unit withdrew after completing the first data entry stage due to staff shortage. In total, the response rate from nurses was 49 % (177/358), and from physicians 53 % (131/245): In the pre-implementation survey, 112 nurses and 82 physicians participated; which is a response rate of 64 % (nurses) and 62 % (physicians). In the post-implementation survey, there were 65 nurses (36 %) and 49 physicians (44 %) who participated. The majority of nurses was female (74.1 % pre, 70.7 % post) with staff position (80.4 % pre, 75.4 % post). Nurses with certified stroke education or another type of advanced education were one-third-in the pre-implementation group and about half of respondents in the post-implementation. The physicians’ group were approximately half female (54.9 % pre, 40.8 % post) with majority on non-management position (staff position) (74.4 % pre, 59.2 % post) and resident level (63.4 % pre, 55.1 % post). All of them represented a wide range of experience from 1 to 43 years of job tenure (Table 2).

(1) Job satisfaction

**Table 2:** Characteristics of participants.

		**Pre-implementation**		**Post-implementation**	
**Nurses**		***n*=112**	**%**	***n*=65**	**%**
Sex	Male	17	15.2	12	18.5
	Female	83	74.1	46	70.7
	No answer	12	10.7	7	10.8
Staff level	Management	9	8.0	7	10.8
	Staff position	90	80.4	49	75.4
	No answer	13	11.6	9	13.8
Professional education	Certified Stroke Nurse, advanced education	40	35.7	36	55.4
Job tenure (years)	Mean, SD, (Min, Max)	7.5, 8.0, (1, 43)		6.9, 5.9, (1, 22)	
**Physicians**		***n*=82**	**%**	***n*=49**	**%**
sex	Male	31	37.8	24	49
	Female	45	54.9	20	40.8
	No answer	6	7.3	5	10.2
Staff level	Management	14	17.1	14	28.6
	Staff position	61	74.4	29	59.2
	No answer	7	8.5	6	12.2
Professional education	Resident	52	63.4	27	55.1
	Specialist	25	30.5	20	40.8
Job tenure (years)	Mean, SD, (Min, Max)	4.4, 5.8, (0, 25)		6.1, 6.5, (0.25, 27)	

Comparing the items regarding global job satisfaction, we found no significant differences from pre-to-post implementation in both the nurses’ and physicians’ group, except in the item “I trust in the hospital management’s ability to manage future challenges”, which from pre to post has decreased in the physicians’ group (*p* = 0.008; Cohen’s d 0.5).

(2) Target parameters

This part contained the symptoms focusing on the target parameters (FeSS) as well as other common nursing problems (BUSS) which were assessed as a control without being subject to an intervention. For all results on these parameters, see the file "supplementary file results Nurses and Doctors en.docx"

### 4.2 Nurses

#### 4.2.1 FeSS-parameters

We found significantly positive effects in terms of perceived self-efficacy and autonomy from pre-to-post surveys, regarding their contribution to managing the FeSS-parameters.

#### 4.2.2 Self-efficacy

In the post-implementation survey, nurses reported on making a very important contribution to the management of fever (*p* < 0.001; Cohens d 0.48) and hyperglycemia (*p* < 0.001; Cohens d 0.56). Nurses reported also to act competently in the management of fever (*p* < 0.001; Cohens d 0.57) and hyperglycemia (*p* = 0.007; Cohens d 0.38). Nurses reported the ability to always respond promptly to changes in blood glucose levels (BGL) (*p* = 0.002; Cohens d 0.44) and dysphagia (*p* = 0.007; Cohens d 0.4).

#### 4.2.3 Autonomy

We found a change in autonomy from pre-to-post, in terms of nurses reporting they could act more independently in fever management (*p* = 0.006; Cohens d 0.42), and dysphagia (*p* = 0.005; Cohens d 0.42) and a gain in competence (knows exactly what to do in case of dysphagia; *p* = 0.007; Cohens d 0.39). In addition, there was significant improvement in reported collaboration by nurses with other nurses (*p* = 0.003; Cohens d 0.45) and specific training on fever following stroke (*p* = 0.006; Cohens d 0.41) (Table 3).

**Table 3:** Nurses’ Results with statistical significance *p* < 0.01 *(*M* = Mean, SD = Standard deviation, *N*=Number of completed answers) For all results see the supplementary file.

			**PRE**			**POST**				
**Symptom**	**Question**	**Item**	M*	**SD**	**N**	**M**	**SD**	**N**	**Cohens d**	***p*-value**
Fever	As nurses,	we make a very important contribution to fever management in the stroke unit.	1.34	0.64	108	1.08	0.32	64	0.48	**<0.001**
Fever	In case of fever in the stroke patient	I can act competently as a nurse.	1.46	0.72	108	1.12	0.33	65	0.57	**<0.001**
Fever	If a stroke patient has elevated temperature (>37.5°),	I can act independently.	1.61	0.89	107	1.26	0.71	65	0.42	**0.006**
Fever	Employees / colleagues are specifically trained regarding fever in case of a stroke	Specialized training	1.53	0.63	107	1.29	0.49	65	0.41	**0.006**
Hyperglycaemia (BGL)	As nurses,	we make a very important contribution to blood glucose management in the stroke unit.	1.39	0.64	109	1.08	0.33	63	0.56	**<0.001**
Hyperglycaemia	As a nurse,	I can act competently in case of elevated BGL in the stroke unit	1.38	0.62	109	1.17	0.38	65	0.38	**0.007**
Hyperglycaemia	If a patient with stroke has elevated BGL,	I can always react promptly.	1.44	0.66	109	1.18	0.43	65	0.44	**0.002**
Dysphagia	If a stroke patient has swallowing disorder/ dysphagia,	I can act independently.	1.91	0.90	109	1.56	0.69	64	0.42	**0.005**
Dysphagia	If a stroke patient has swallowing disorder/ dysphagia,	I can always react promptly.	1.73	0.82	108	1.43	0.61	65	0.40	**0.007**
Dysphagia	If a stroke patient has swallowing disorder/ dysphagia,	I know exactly what needs to be done.	1.61	0.77	109	1.34	0.51	65	0.39	**0.007**
	Rating the cooperation within the nursing staff		2.06	0.94	103	1.67	0.72	63	0.45	**0.003**

#### 4.2.4 BUSS-parameters

Regarding the control items in terms of other common nursing problems (BUSS), there were no significant changes from pre-to-post survey.

### 4.3 Physicians

#### 4.3.1 FeSS parameters

In the medical profession, significant improvements were reported in training activities to introduce new medical colleagues regarding FeSS parameters fever (*p* < 0.001; Cohens d 0.59) and dysphagia (*p* < 0.001; Cohens d 0.64). No significant (*p* < 0.01) changes were shown regarding the FeSS parameters.

#### 4.3.2 BUSS parameters

Interestingly, we noted effects in the medical profession on parameters where no intervention was been implemented (Table 4). In contrast to the nurses’ group, in the control items section (BUSS), physicians reported significant changes in training activities for restlessness (*p* < 0.001; Cohens d 0.76), and sleep disorders (*p* < 0.001; Cohens d 0.79).

**Table 4:** Physicians results with statistical significance (*p* < 0.01) *(*M* = Mean, SD = Standard deviation, *N*=Number of completed answers). For all results see the supplementary file.

	Physicians		PRE			POST				
**Symptom**	**Item**	**Cluster**	**Mean**	**SD**	**n**	**Mean**	**SD**	**n**	**Cohens d**	***p*-value**
Sleep disorders	If a stroke patient has sleep disorders	…... I feel competent in working with the nursing staff.	2.14	0.93	81	1.68	0.59	47	0.55	**0.001**
Sleep disorders	If a stroke patient has sleep disorders	… always optimal care for the patient is guaranteed.	2.44	0.96	78	1.98	0.77	47	0.51	**0.004**
Sleep disorders	If a stroke patient has sleep disorders	… I regularly discuss the findings with the nursing staff.	2.18	0.95	80	1.77	0.76	47	0.46	**0.009**
Sleep disorders	In case of sleep disorders in the stroke unit, I feel the nursing staff as competent	Nurses can act competently (from the doctors' point of view)	2.41	0.93	81	2.00	0.80	48	0.46	**0.01**
Restlessness	If a stroke patient is very restless,	… cooperation with the nursing staff is very well organized.	2.28	1.02	80	1.81	0.64	48	0.52	**0.002**
Restlessness	If a stroke patient is very restless,	… always optimal care for the patient is guaranteed.	2.53	1.00	79	2.06	0.84	48	0.50	**0.005**
Specialized training (targeted induction)	Regarding **restlessness** in case of stroke,	employees/colleagues are specifically trained.	2.23	0.64	80	1.73	0.68	48	0.76	**<0.001**
Specialized training (targeted induction)	Regarding **sleep disorders** in case of stroke,	employees/colleagues are specifically trained.	2.64	0.58	80	2.11	0.81	47	0.79	**<0.001**

Physicians reported to feel more competent in working with the nursing staff in case of sleep disorders (*p* = 0.001; Cohens d 0.55) and discussed their findings with the nursing staff (*p* = 0.009; Cohens d 0.46). The doctors reported that if a stroke patient has sleep disorders, always optimal care for the patient were guaranteed (*p* = 0.004; Cohens d 0.51) and attributed the nurses in acting competently (*p* = 0.01; Cohens d 0.46).

Regarding the nursing phenomena of restlessness, there seems to be raised awareness concerning restlessness in cooperation with nursing staff (*p* = 0.002; Cohens d 0.52) and quality of care (*p* = 0.005; Cohens d 0.5). On blood pressure and pain management, there were no significant changes.

#### 4.3.3 Additional comments

An additional field collected free comments (“What do you take away from the project?”). A few participants used this field to write up additional thoughts reflecting the project (Table 5).

**Table 5:** Additional comments to the project.

**Nurses’ comments**	**Category**	**Doctors’ comments**	**Category**
Self confidence in the own responsibility has improved among colleagues. Priorities in caring for stroke patients have changed. I think some colleagues have learned that care is not only basic care, but unfortunately not all of them. E10	Satisfaction with the professional role		
Raising awareness of the importance of controlling vital signs in a stroke unit How important autonomy is in care and how it can structure and simplify work processes H15	Awareness Satisfaction	Significant improvement in cooperation between nursing staff and doctors in important areas of patient care (blood sugar, blood pressure, dysphagia, restlessness, sleep dis-orders). More responsibility and greater appreciation for nursing. A3	Interprofessional communication Nurses’ autonomy
Faster decisions, (…) fewer calls to the doctor. Enhancement of the nursing work (…) to react even faster to the patient's condition. Work satisfaction also increases H16	Autonomy Satisfaction	There can never be enough communication between those involved in the therapy. A5	Interprofessional communication
Better care for patients. Independent work, accountability and quality improvement B2	Autonomy	Participation has led to an improvement in quality. The perception of a multi-professional team is strengthened. A2	Interprofessional communication
Was interesting to work independently and with more responsibility. H13	Autonomy		
Independence - I can decide more quickly B3	Autonomy	Autonomous action by nursing staff relieves the burden on the medical staff and promotes the satisfaction of nursing staff. A7	Satisfaction Nurses’ autonomy
More autonomy in decision-making must be desired by each individual. E9	Autonomy Ambiguity		
I appreciate the more autonomous way. Some employees still moan about additional workloads, but these are compensated for by the time saved elsewhere. H14	Autonomy Ambiguity	The concept of taking over medical activities was regulated before, so there was only an innovation in the swallowing test. The nursing staff initially accepted the swallowing test reluctantly, but it is now routine. A4	Ambiguity

Amongst nurses, we noted higher awareness, more autonomy, satisfaction with the project and the professional role, but also ambiguity amongst some nurses regarding the increased responsibility. Physicians appreciated the increased autonomy of nurses, improved interprofessional communication and collaboration as well as improved care, but also noted the ambiguity of some nurses about nurses increased autonomy.

## 5 Discussion

The aim of our study was to explore whether the implementation of the nurse-initiated FeSS Protocols would improve nurses’ job satisfaction and autonomy, and physicians’ perception of nurse autonomy. We focused on perceived autonomy and self-efficacy, relieving nurses and physicians from having to discuss each individual patient order for managing fever and hyperglycemia.

There was no change between pre-and-post implementation with global job satisfaction. The only alteration out of 55 items from pre-to-post was physicians reporting “less confidence into the hospital management’s ability to manage future challenges”. This might be due to general challenges in German health politics, but also to the disruptions to clinical care caused by the COVID-19 pandemic at the time of the survey. Several pandemic waves affected processes and workflows in hospitals during the study. They had high impact on the staff workload and might have caused staff exhaustion in many aspects (Schug et al., 2022; Hering et al., 2022).

Part two of the questionnaire contained newly designed questions, customized to focus on stroke care. In the nursing profession, our results showed there have been highly significant changes (*p* < 0.01) in the ability to manage the FeSS parameters autonomously in Germany. The data from our survey show that nurses reported they were able to act in a timelier and more competent manner and stated they made a very important contribution to fever and hyperglycemia management as well as the dysphagia management in the stroke unit. As a side effect, the collaboration within the nursing staff has improved (*p* = 0.003). The additional comments supported these results, with nurses stating raised awareness on the advantage of them having more autonomy (Table 5).

The FeSS parameters were the subject of our implementation activities. In addition, as a control question, we asked about other common nursing activities. These activities were the management of blood pressure, restlessness, pain, and sleep disorder (BUSS). Regarding these parameters, we expected no change, since no training was provided. Interestingly, the effect of implementing the FeSS Protocol generated unexpected effects, whereby enhanced competencies extended to other nurses’ tasks that are similarly relevant, but were not subject to the intervention. This concept of ‘spillover’ which “describes the boundary between different roles and worlds as permeable” (DeMarco, 2002) might explain these unexpected effects we see in the control parameters: Nurses reported feeling more self-confident in managing blood pressure (Cohens d 0.37; *p* = 0.012) which is worth noting for its clinical significance.

The same phenomenon was observed in the physician’s group. In the medical profession, the physicians significantly intensified their activities to introduce new team members into stroke care more thoroughly. Not only the training regarding FeSS Protocols, but also training regarding restlessness and sleep disorders was perceived to be improved. The medical practitioners also reported improved interprofessional communication (*p* = 0.009, Cohens d 0.46), as well as improved awareness regarding diagnosis of patient restlessness (*p* = 0.002, Cohens d 0.52). A physician mentioned this in an additional comment, naming also the BUSS parameters (Table 4) as important areas of patient care where they noted significant improvements in cooperation between nursing staff and doctors, deducing more responsibility and appreciation for nursing care (A3, Table 5). The FeSS Protocols also appeared to be working as a ‘spillover agent’, as physicians reported the nurses to be more competent and transferred this perception of competency from FeSS to BUSS. Another physician added in an additional comment (A7, Table 5), that autonomous action by nursing staff would relieve the burden on the medical service and promote the satisfaction of nursing staff. Thus, the physicians definitely perceived the gain in competence as an advantage of the project.

The findings support the widely known relevance of the multi-faceted nurses’ roles on the stroke unit (Weir and Cadilhac, 2007; Langhorne and Ramachandra, 2020) and show the power of the FeSS Protocols implementation to change professional practice and to improve nursing care as perceived by both, medical and nursing staff. As QASC Germany demonstrated clinician behavior changes in adherence to the FeSS Protocol (Cassier-Woidasky et al., 2024) as also demonstrated in the QASC Europe Study (Middleton et al., 2023), the current results support the relevance of multifaceted implementation processes for staff satisfaction. In addition to the QASC Europe process evaluation (McInnes et al., 2024), our study supports the importance of defining and establishing nurses’ roles in stroke care. As further effort is needed to optimize the adherence to the FeSS Protocols (Ding et al., 2024), our results emphasize the need to consider self-efficacy and satisfaction during the implementation process.

Moreover, using the FeSS Protocols has increased nurses’ self-efficacy demonstrating that legal reporting requirements may be insufficient alone to drive quality improvement. This DRG reimbursement system, implemented to improve the allocation, requires health professionals to document each procedure performed on the patient, but it does not improve quality of care, instead, it has significant negative impact on nurses’ satisfaction and the nurse-perceived quality of care (Zander et al., 2013b).

### 5.1 Implications

For future practice to improve professional nursing care in Germany, we propose to implement stroke-specific Standard Operating Procedures, not only the FeSS Protocols, but also guidelines for nurses to manage other evidence-based nursing activities more autonomously. However, as international research on FeSS implementation has revealed, sustainable adherence on the Protocols requires longer-term processes and activities (Coughlan et al., 2024).

It seems surprising that a comparatively small intervention that was introduced during the pandemic under difficult conditions in turbulent times has proven to have an effect later on. This is an indicator that we should also take a closer look at small interventions in other areas and design routines that make work easier and increase job satisfaction.

These findings might also support the demand for advanced nurses’ roles based on expanded academic education as they are common in most parts of the world (Schober and Affara, 2008). The need for these improvements is illustrated by the additional comments by the nurses (Table 5): On the one hand nurses greatly appreciated working more autonomously, while on the other hand, other nurses were ambiguous regarding taking on more responsibility and accountability. It is an important part of staff satisfaction (Zander, 2017), but requires systematic improvements in advanced nursing education to the tertiary sector and has been demanded for decades also for Germany by scientific advisory boards like WISSENSCHAFTSRAT 2012, Wissenschaftsrat, 2023.

### 5.2 Limitations

Our study faced several limitations on account of the extended project time caused by the COVID-19 pandemic. Due to pandemic related restrictions and management decisions as well as high staff turnover rates reported by site champions, staff members faced high workload and exhaustion, which may have affected the post-test survey response rates, however our responses rates exceeded those previously reported between 5 and 40 % (Döring and Bortz, 2016). So, it would be desirable to repeat this survey after implementing the FeSS Protocols in a larger entity of stroke units. Methodologically, our one-group pre-to-post-test design was a known limitation. Acquiring a control group was not feasible, but this would enable us to determine more precisely whether changes in the dependent variable are due to the intervention or to external factors. We did aim to control this by measuring the BUSS parameter and the general satisfaction as controls, but replication would be desirable to confirm this finding.

## 6 Conclusions

The nurse-initiated FeSS Protocols made an important contribution not only to the nurses’ professional self-concept and self-confidence and the perception of nurses as competent team members. Even when strict documentation policies attempt to guarantee high quality care, the mere existence of a directive like the remuneration requirements does not necessarily improve care. Despite well-established therapy guidelines and tightly regulated quality control, the transfer of the FeSS Protocols to German stroke units revealed potential for improvement by improving autonomy for nurses as a regular part of nurses’ work clinical practice, as it is in other countries of the world (Schober and Affara, 2008). This would not only improve the quality of care, but could also contribute to increase attractiveness of work at the stroke unit.

## Funding

This work was supported by a non-restricted educational research grant from Angels Initiative (Boehringer Ingelheim). The funder had no role in any part of the study.

## Data availability

The datasets used and analysed during the current study are available from the corresponding author on reasonable request.

## CRediT authorship contribution statement

**Anne-Kathrin Cassier-Woidasky:** Writing – review & editing, Writing – original draft, Project administration, Investigation, Funding acquisition, Formal analysis, Conceptualization. **Winfried Zinn:** Writing – review & editing, Software, Resources, Methodology, Formal analysis, Data curation, Conceptualization. **Sandy Middleton:** Writing – review & editing, Supervision, Resources, Funding acquisition, Conceptualization. **Simeon Dale:** Writing – review & editing, Supervision, Resources, Project administration, Conceptualization. **Waltraud Pfeilschifter:** Writing – review & editing, Investigation, Funding acquisition, Formal analysis, Conceptualization.

## Declaration of competing interest

A.K.C.W., S.M., S.D., W.P. none. W.Z. had a subcontract as consultant to develop the questionnaire and to perform the statistics.

## Appendix Supplementary materials

Image, application 1
