# Supplementary material for: 'Do Fever-Sugar-Swallow Protocols improve nurses' and physicians' satisfaction with the management of fever, hyperglycemia and dysphagia in stroke patients: A pre-and post-implementation survey of the “Quality in Acute Stroke Care” (QASC) Program‘
Source: Int J Nurs Stud Adv. 2025 Jun 30;9:100374. doi: 10.1016/j.ijnsa.2025.100374 (PMC12275883; doi:10.1016/j.ijnsa.2025.100374)
Supplement: Supplementary file 1 [file mmc1.docx]

| **BG**  **code** | **item** | **Question text** | **Symptom** | **Cluster** | **Mea n** | **SD** | **N** | **M** | **SD** | **N** | **Cohen' s d** | **p- value** |
| --- | --- | --- | --- | --- | --- | --- | --- | --- | --- | --- | --- | --- |
| 102 | E520 | Grade for cooperation with physiotherapists |  |  | 1,86 | 0,93 | 111 | 1,75 | 0,79 | 65 | 0,12 | 0,441 |
| 102 | E521 | Grade for cooperation with medical service |  |  | 2,35 | 0,95 | 110 | 2,31 | 0,92 | 65 | 0,05 | 0,748 |
| 102 | E522 | Grade for cooperation with nursing service |  |  | 2,06 | 0,94 | 103 | 1,67 | 0,72 | 63 | 0,45 | 0,003 |
| 102 | E523 | Grade for cooperation with speech therapists/speech therapists |  |  | 1,81 | 0,84 | 111 | 1,82 | 0,93 | 65 | 0,01 | 0,974 |
| 102 | E524 | Grade for cooperation with occupational therapists |  |  | 1,85 | 0,85 | 110 | 1,78 | 0,76 | 65 | 0,09 | 0,576 |
| 102 | R01 | a. In my area, employees / colleagues are specifically trained. |  |  | 1,73 | 0,61 | 108 | 1,68 | 0,62 | 65 | 0,09 | 0,571 |
| 102 | E503 | e. Employees / colleagues are specifically trained on the topic of  fever in stroke. |  | Targeted training | 1,53 | 0,63 | 107 | 1,29 | 0,49 | 65 | 0,41 | 0,006 |
| 102 | E501 | c. Employees / colleagues are specifically trained on the topic of  blood sugar in stroke. |  | Targeted training | 1,57 | 0,66 | 106 | 1,34 | 0,51 | 65 | 0,37 | 0,012 |
| 102 | E505 | b./g. Employees / colleagues are specifically trained on the topic of  dysphagia in stroke patients. |  | Targeted training | 1,42 | 0,52 | 106 | 1,28 | 0,48 | 65 | 0,29 | 0,061 |
| 102 | E504 | f. Employees / colleagues are specifically trained on the topic of  blood pressure in the event of a stroke. |  | Targeted training | 1,36 | 0,56 | 107 | 1,23 | 0,46 | 65 | 0,26 | 0,090 |
| 102 | E500 | b. Employees / colleagues are specifically trained on the topic of pain  in stroke patients. |  | Targeted training | 2,09 | 0,78 | 104 | 1,91 | 0,73 | 64 | 0,24 | 0,131 |
| 102 | E506 | h. Employees / colleagues are specifically trained on the topic of  restlessness in the event of a stroke. |  | Targeted training | 1,92 | 0,76 | 106 | 1,94 | 0,79 | 65 | 0,03 | 0,849 |
| 102 | E502 | d. Employees / colleagues are specifically trained on the topic of  sleep in stroke patients. |  | Targeted training | 2,17 | 0,75 | 103 | 2,06 | 0,81 | 65 | 0,15 | 0,364 |
| 102 | E52 | If a patient with a stroke has an elevated temperature (>37.5°), then ...  I know exactly what to do. | Fever | ... I know exactly what to do. | 1,22 | 0,50 | 108 | 1,08 | 0,27 | 65 | 0,34 | 0,014 |
| 102 | E53 | If a patient with a stroke has an elevated temperature (>37.5°), then ...  I can always react promptly. | Fever | ... I can always react promptly. | 1,43 | 0,67 | 108 | 1,22 | 0,54 | 65 | 0,34 | 0,026 |
| 102 | E54 | If a patient with a stroke has an elevated temperature (>37.5°), then ...  I can act independently. | Fever | ... I can act independently. | 1,61 | 0,89 | 107 | 1,26 | 0,71 | 65 | 0,42 | 0,006 |
| 102 | E55 | If a patient with a stroke has an elevated temperature (>37.5°), then ...  I feel competent in working with the doctors. | Fever | ... I feel competent in working with the doctors. | 1,62 | 0,84 | 107 | 1,41 | 0,66 | 64 | 0,27 | 0,071 |
| 102 | E56 | If a patient with a stroke has an elevated temperature (>37.5°), then ...  I must ask the doctors regularly for substantive reasons. | Fever | ... I must ask the doctors regularly for substantive reasons. | 3,36 | 1,36 | 107 | 3,60 | 1,38 | 65 | 0,18 | 0,258 |
| 102 | E58 | If a patient with a stroke has an elevated temperature (>37.5°), then ...  I regularly must obtain an order, although I would know what to do. | Fever | ... I must obtain an order regularly, even though I  know what to do. | 3,05 | 1,48 | 107 | 3,40 | 1,48 | 65 | 0,24 | 0,130 |
| 102 | E60 | If a patient with a stroke has an elevated temperature (>37.5°), then ...  cooperation with the doctors is very well organized. | Fever | ... cooperation with the doctors/nursing staff is very well organized. | 1,95 | 1,00 | 105 | 1,83 | 0,94 | 65 | 0,12 | 0,427 |
| 102 | E61 | If a patient with a stroke has an elevated temperature (>37.5°), then ...  I know when to call in other people for support if necessary. | Fever | ... I know when to call in other people for support if  necessary. | 1,49 | 0,85 | 107 | 1,31 | 0,56 | 65 | 0,24 | 0,099 |
| 102 | E62 | If a patient with a stroke has an elevated temperature (>37.5°), then ...  optimal care for the patient is always guaranteed. | Fever | ... optimal care of the patient is always guaranteed. | 1,79 | 0,90 | 108 | 1,66 | 0,80 | 65 | 0,15 | 0,340 |
| 102 | E63 | As a nurse, I can act competently in the event of fever in the stroke  unit. | Fever | Nurse can act competently (from the perspective of  the nursing staff) | 1,46 | 0,72 | 108 | 1,12 | 0,33 | 65 | 0,57 | 0,000 |
| 102 | E65 | We nurses make a very important contribution to fever management in  the stroke unit. | Fever | Nursing staff make a very important contribution (from the nursing staff's perspective) | 1,34 | 0,64 | 108 | 1,08 | 0,32 | 64 | 0,48 | 0,000 |
| 102 | E18 | If a patient with a stroke has elevated blood sugar levels, then ... I know  exactly what to do. | Blood sugar | ... I know exactly what to do. | 1,29 | 0,58 | 109 | 1,14 | 0,39 | 65 | 0,30 | 0,037 |
| 102 | E19 | If a patient with a stroke has elevated blood sugar levels, then ... I can  always react promptly. | Blood sugar | ... I can always react promptly. | 1,44 | 0,66 | 109 | 1,18 | 0,43 | 65 | 0,44 | 0,002 |
| 102 | E20 | If a patient with a stroke has elevated blood sugar levels, then ... I can  act independently. | Blood sugar | ... I can act independently. | 1,56 | 0,87 | 109 | 1,38 | 0,78 | 65 | 0,21 | 0,173 |
| 102 | E21 | If a patient with a stroke has elevated blood sugar levels, then ... I feel  competent in working with the doctors. | Blood sugar | ... I feel competent in working with the doctors. | 1,64 | 0,86 | 109 | 1,49 | 0,73 | 65 | 0,18 | 0,222 |
| 102 | E22 | If a patient with a stroke has elevated blood sugar levels, then ... I have  to ask the doctors regularly for substantive reasons. | Blood sugar | ... I must ask the doctors regularly for substantive reasons. | 3,53 | 1,27 | 105 | 3,42 | 1,30 | 65 | 0,09 | 0,563 |
| 102 | E24 | If a patient with a stroke has elevated blood sugar levels, then ... I  regularly must ask for an order, even though I know what to do. | Blood sugar | ... I must obtain an order regularly even though I  know what to do. | 3,18 | 1,34 | 107 | 3,42 | 1,41 | 64 | 0,18 | 0,267 |
| 102 | E26 | If a patient with a stroke has elevated blood sugar levels, then ...  cooperation with the doctors is very well organized. | Blood sugar | ... cooperation with the doctors is very well organized. | 2,09 | 1,04 | 108 | 2,08 | 1,00 | 64 | 0,01 | 0,928 |
| 102 | E27 | If a patient with a stroke has elevated blood glucose levels, then ... I  know when to call in other people for support if necessary. | Blood sugar | ... I know when to call in other people for support if  necessary. | 1,47 | 0,70 | 108 | 1,31 | 0,53 | 65 | 0,26 | 0,083 |
| 102 | E28 | If a patient with a stroke has elevated blood sugar levels, then ...  optimal care for the patient is always guaranteed. | Blood sugar | ... optimal care for the patient is always guaranteed. | 1,84 | 0,86 | 108 | 1,68 | 0,73 | 65 | 0,20 | 0,178 |
| 102 | E29 | As a nurse, I can act competently in the event of elevated blood  glucose levels in the stroke unit. | Blood sugar | Nurse can act competently (from the perspective of  the nursing staff) | 1,38 | 0,62 | 109 | 1,17 | 0,38 | 65 | 0,38 | 0,007 |
| 102 | E31 | We nursing staff make a very important contribution to blood glucose  management in the stroke unit. | Blood sugar | Nursing staff make a very important contribution  (from the nursing staff's perspective) | 1,39 | 0,64 | 109 | 1,08 | 0,33 | 63 | 0,56 | 0,000 |
| 102 | E33 | The contribution of nursing care to blood glucose management in the  stroke unit is perceived as important by the physicians. | Blood  sugar | Contribution of nursing staff is perceived by doctors (from the nursing staff's perspective) | 1,92 | 1,05 | 105 | 1,87 | 0,93 | 62 | 0,05 | 0,737 |
| 102 | E34 | The procedure for elevated blood glucose levels is first class. | Blood sugar | Procedure for symptoms is first class | 1,94 | 0,86 | 109 | 1,69 | 0,75 | 64 | 0,30 | 0,049 |
| 102 | E86 | If a patient with dysphagia comes to the stroke unit, then ... I know  exactly what to do regarding dysphagia. | Dysphagia | ... I know exactly what to do. | 1,61 | 0,77 | 109 | 1,34 | 0,51 | 65 | 0,39 | 0,007 |
| 102 | E87 | If a patient with a swallowing disorder/ dysphagia comes to the stroke  unit, then ... I can always react promptly. | Dysphagia | ... I can always react promptly. | 1,73 | 0,82 | 108 | 1,43 | 0,61 | 65 | 0,40 | 0,007 |
| 102 | C01 | If a patient with a swallowing disorder/ dysphagia comes to the stroke  unit, then ... I can act independently. | Dysphagia | ... I can act independently. | 1,91 | 0,90 | 109 | 1,56 | 0,69 | 64 | 0,42 | 0,005 |
| 102 | C02 | If a patient with a swallowing disorder/ dysphagia comes to the stroke  unit, then I feel competent in working with the doctors. | Dysphagia | ... I feel competent in the cooperation with the doctors. | 1,96 | 0,89 | 108 | 1,89 | 0,92 | 65 | 0,08 | 0,621 |
| 102 | C03 | If a patient with a swallowing disorder/ dysphagia comes to the stroke  unit, then ... I regularly discuss the findings with the doctors. | Dysphagia | ... I regularly discuss the findings with the nursing staff. | 2,15 | 1,10 | 108 | 2,05 | 1,05 | 64 | 0,09 | 0,548 |
| 102 | C04 | If a patient with dysphagia comes to the stroke unit, then ... I regularly must wait for a doctor's order, even though I am confident that I can  assess the patient's ability to swallow. | Dysphagia | ... I regularly must obtain an order, although I would know what to do. | 3,31 | 1,27 | 108 | 3,77 | 1,13 | 65 | 0,37 | 0,015 |
| 102 | C06 | If a patient with dysphagia is admitted to the stroke unit, then ...  cooperation with the doctors is very well organized | Dysphagia | ... cooperation with the doctors is very well organized. | 2,33 | 1,01 | 107 | 2,20 | 0,92 | 65 | 0,13 | 0,399 |
| 102 | C07 | If a patient with a swallowing disorder/ dysphagia comes to the stroke  unit, then ... I must ask the doctors regularly for reasons of content. | Dysphagia | ... I must ask the doctors regularly for  substantive reasons. | 3,37 | 1,23 | 105 | 3,43 | 1,32 | 65 | 0,05 | 0,771 |
| 102 | C08 | If a patient with a swallowing disorder/ dysphagia comes to the stroke unit, then ... I feel competent in working with the speech  therapists/speech therapists/nurses. | Dysphagia | ... I feel competent in working with speech therapists/speech therapists/nurses. | 1,68 | 0,86 | 109 | 1,66 | 0,80 | 65 | 0,02 | 0,893 |
| 102 | C09 | If a patient with a swallowing disorder/ dysphagia comes to the stroke unit, then ... I must regularly consult with the speech and language therapist, although I am confident that I can assess the swallowing  ability. | Dysphagia | ... I must consult the speech and language therapist regularly, although I am confident that I can assess the swallowing ability. | 2,83 | 1,23 | 108 | 3,18 | 1,14 | 65 | 0,29 | 0,060 |
| 102 | C11 | If a patient with a swallowing disorder/ dysphagia is admitted to the stroke unit, then ... I regularly discuss the findings with speech therapists/speech therapists //... I only receive regular queries from the  nursing staff if this is necessary. | Dysphagia | ... I regularly discuss the findings with the speech and language therapists //... I only receive regular queries from the nursing staff if this is necessary. | 1,66 | 0,99 | 107 | 1,63 | 0,72 | 65 | 0,04 | 0,803 |
| 102 | C12 | If a patient with a swallowing disorder/ dysphagia comes to the stroke unit, then cooperation with the speech therapists/ speech  therapists/ nursing staff is very well organized | Dysphagia | .... Cooperation with speech therapists/ speech therapists/ nursing staff is very well organized | 1,69 | 0,94 | 108 | 1,80 | 0,83 | 65 | 0,12 | 0,444 |
| 102 | U14 | If a patient with dysphagia comes to the stroke unit, then ... I know  when to call in other people for support if necessary. | Dysphagia | ... I know when I need to call in other people for support if necessary. | 1,46 | 0,76 | 105 | 1,40 | 0,63 | 65 | 0,08 | 0,597 |
| 102 | U15 | If a patient with a swallowing disorder/ dysphagia is admitted to the  stroke unit, then ... optimal care of the patient is always guaranteed. | Dysphagia | ... optimal care of the patient is always guaranteed. | 1,85 | 0,86 | 106 | 1,75 | 0,71 | 65 | 0,12 | 0,433 |
| 102 | U17 | I can act competently as a nurse when assessing dysphagia in the  stroke unit. | Dysphagia | Nurse can act competently (from the perspective of  the nursing staff) | 1,72 | 0,76 | 108 | 1,46 | 0,56 | 65 | 0,38 | 0,011 |
| 102 | U18 | We nurses make a very important contribution to the assessment of  dysphagia in the stroke unit. | Dysphagia | Nurses make a very important contribution (from  the nurses' perspective) | 1,56 | 0,71 | 108 | 1,32 | 0,56 | 65 | 0,35 | 0,019 |
| 102 | U20 | In the assessment of dysphagia in the stroke unit, the contribution of  nursing is perceived as important by the physicians. | Dysphagia | Contribution of nursing staff is perceived by  physicians (from the nursing staff's perspective) | 2,04 | 0,98 | 106 | 2,02 | 0,93 | 64 | 0,02 | 0,883 |
| 102 | U23 | In the assessment of dysphagia on the stroke unit, the contribution of nursing care is perceived as important by speech and language  therapists. | Dysphagia | Contribution of nursing staff is perceived by speech therapists (from the nursing staff's perspective) | 2,04 | 0,99 | 100 | 2,08 | 0,93 | 60 | 0,04 | 0,781 |
| 102 | U29 | The procedure for suspected dysphagia is first class. | Dysphagia | Procedure for symptoms is first class | 1,99 | 0,89 | 108 | 1,86 | 0,75 | 65 | 0,15 | 0,308 |
| 102 | E69 | If a patient with a stroke has high blood pressure, then ... I know  exactly what to do. | Blood  pressure | ... I know exactly what to do. | 1,29 | 0,60 | 109 | 1,20 | 0,41 | 64 | 0,17 | 0,239 |
| 102 | E70 | If a patient with a stroke has elevated blood pressure, then ... I can  always react promptly. | Blood  pressure | ... I can always react promptly. | 1,40 | 0,63 | 107 | 1,36 | 0,65 | 64 | 0,07 | 0,676 |
| 102 | E71 | If a patient with a stroke has elevated blood pressure, then ... I can act  independently. | Blood  pressure | ... I can act independently. | 1,72 | 0,92 | 109 | 1,63 | 0,87 | 62 | 0,10 | 0,543 |
| 102 | E72 | If a patient with a stroke has high blood pressure, then ... I feel  competent in working with the doctors. | Blood  pressure | ... I feel competent in working with the doctors. | 1,68 | 0,81 | 107 | 1,56 | 0,76 | 62 | 0,15 | 0,345 |
| 102 | E73 | If a patient with a stroke has elevated blood pressure values, then ... I  must ask the doctors regularly for substantive reasons. | Blood  pressure | ... I must ask the doctors regularly for substantive reasons. | 3,29 | 1,24 | 107 | 3,38 | 1,28 | 64 | 0,07 | 0,671 |
| 102 | E75 | If a patient with a stroke has elevated blood pressure, then ... I  regularly must ask for an order, even though I know what to do. | Blood  pressure | ... I must obtain an order regularly, although I  would know what to do. | 2,92 | 1,34 | 109 | 2,92 | 1,42 | 64 | 0,00 | 0,984 |
| 102 | E77 | If a patient with a stroke has elevated blood pressure values, then ...  cooperation with the doctors is very well organized. | Blood  pressure | ... cooperation with the doctors is very well organized. | 1,90 | 0,93 | 109 | 1,92 | 0,88 | 64 | 0,02 | 0,872 |
| 102 | E78 | If a patient with a stroke has high blood pressure, then ... I know when  to call in other people for support if necessary. | Blood  pressure | ... I know when to call in other people for support if  necessary. | 1,43 | 0,73 | 106 | 1,28 | 0,49 | 64 | 0,23 | 0,104 |
| 102 | E79 | If a patient with a stroke has elevated blood pressure values, then ...  optimal care for the patient is always guaranteed. | Blood  pressure | ... optimal care for the patient is always guaranteed. | 1,72 | 0,78 | 109 | 1,78 | 0,83 | 64 | 0,07 | 0,658 |
| 102 | E80 | As a nurse, I can act competently in the event of elevated blood  pressure values in the stroke unit. | Blood  pressure | Nurse can act competently (from the perspective of  the nursing staff) | 1,45 | 0,70 | 109 | 1,22 | 0,49 | 64 | 0,37 | 0,012 |
| 102 | E82 | We nurses make a very important contribution to blood pressure  management in the stroke unit. | Blood  pressure | Nursing staff make a very important contribution  (from the nursing staff's perspective) | 1,34 | 0,60 | 109 | 1,14 | 0,43 | 63 | 0,36 | 0,014 |
| 102 | E84 | The contribution of nursing care to blood pressure management in the  stroke unit is perceived as important by the physicians. | Blood  pressure | Contribution of nursing staff is perceived by doctors (from the nursing staff's perspective) | 1,81 | 0,89 | 105 | 1,65 | 0,93 | 62 | 0,18 | 0,263 |
| 102 | E85 | The procedure for elevated blood pressure values is first class. | Blood  pressure | Procedure for symptoms is first class | 1,79 | 0,83 | 107 | 1,69 | 0,77 | 64 | 0,13 | 0,397 |
| 102 | E03 | If a patient with a stroke has severe pain, then ... I can act  independently. | Pain | ... I can act independently. | 2,13 | 1,00 | 110 | 1,98 | 1,12 | 65 | 0,14 | 0,400 |
| 102 | E02 | If a patient with a stroke has severe pain, then ... I can always react  promptly. | Pain | ... I can always react promptly. | 1,74 | 0,71 | 110 | 1,51 | 0,66 | 65 | 0,33 | 0,034 |
| 102 | E04 | If a patient with a stroke has severe pain, then ... I feel competent in  working with the doctors. | Pain | ... I feel competent in working with the doctors. | 1,93 | 0,93 | 109 | 1,66 | 0,82 | 65 | 0,30 | 0,051 |
| 102 | E05 | If a patient with a stroke has severe pain, then ... I must ask the  doctors regularly for substantive reasons. | Pain | ... I must ask the doctors regularly for substantive reasons. | 3,25 | 1,23 | 106 | 3,31 | 1,20 | 65 | 0,05 | 0,744 |
| 102 | E01 | If a patient with a stroke has severe pain, then ... I know exactly what  to do. | Pain | ... I know exactly what to do. | 1,59 | 0,68 | 110 | 1,37 | 0,57 | 65 | 0,34 | 0,023 |
| 102 | E07 | If a patient with a stroke has severe pain, then ... I regularly must ask  for an order, even though I know what to do. | Pain | ... I regularly must obtain an order, although I would know what to do. | 2,65 | 1,41 | 108 | 2,52 | 1,38 | 65 | 0,09 | 0,568 |
| 102 | E09 | If a patient with a stroke has severe pain, then ... cooperation with the  doctors is very well organized. | Pain | ... cooperation with the doctors is very well organized. | 2,35 | 0,99 | 110 | 2,42 | 1,05 | 64 | 0,07 | 0,678 |
| 102 | E10 | If a patient with a stroke has severe pain, then ... I know when to call in  other people for support if necessary. | Pain | ... I know when to call in other people for support if  necessary. | 1,48 | 0,74 | 107 | 1,31 | 0,56 | 65 | 0,25 | 0,092 |
| 102 | E11 | If a patient with a stroke has severe pain, then ... optimal care for the  patient is always guaranteed. | Pain | ... optimal care for the patient is always guaranteed. | 2,17 | 0,89 | 109 | 2,12 | 0,86 | 65 | 0,06 | 0,708 |
| 102 | E12 | As a nurse, I can act competently in pain management on the stroke  unit. | Pain | Nurse can act competently (from the perspective of  the nursing staff) | 1,94 | 0,91 | 109 | 1,89 | 0,88 | 63 | 0,06 | 0,692 |
| 102 | E14 | We nurses make a very important contribution to pain management in  the stroke unit. | Pain | Nursing staff make a very important contribution  (from the nursing staff's perspective) | 1,61 | 0,86 | 109 | 1,38 | 0,55 | 63 | 0,29 | 0,039 |
| 102 | E16 | The contribution of nursing care to pain management in the stroke unit  is perceived as important by the doctors. | Pain | Contribution of nursing staff is perceived by doctors (from the nursing staff's perspective) | 2,21 | 1,10 | 108 | 2,28 | 0,97 | 61 | 0,06 | 0,688 |
| 102 | E17 | Our pain management is first class. | Pain | Symptom process is first class | 2,66 | 1,18 | 105 | 2,54 | 0,95 | 63 | 0,11 | 0,479 |
| 102 | U30 | If a patient with a stroke is very restless, then ... I know exactly what to  do. | Restlessness | ... I know exactly what to do. | 1,97 | 0,85 | 107 | 1,89 | 0,89 | 64 | 0,09 | 0,559 |
| 102 | U31 | If a patient with a stroke is very restless, then ... is always responded to  promptly. | Restlessness | ... I can always react promptly. | 2,13 | 0,91 | 106 | 2,00 | 0,89 | 64 | 0,15 | 0,354 |
| 102 | U32 | If a patient with a stroke is very restless, then ... I can react  independently. | Restlessnes  s | ... I can act independently. | 2,62 | 1,12 | 104 | 2,65 | 1,14 | 63 | 0,03 | 0,845 |
| 102 | U33 | If a patient with a stroke is very restless, then ... I feel competent in  working with the doctors. | Restlessnes  s | ... I feel competent in working with the doctors. | 2,15 | 0,96 | 105 | 2,19 | 1,08 | 62 | 0,04 | 0,805 |
| 102 | U34 | If a patient with a stroke is very restless, then ... I must ask the  doctors regularly for substantive reasons. | Restlessnes  s | ... I must ask the doctors regularly for substantive reasons. | 3,21 | 1,23 | 106 | 2,92 | 1,19 | 64 | 0,24 | 0,136 |
| 102 | U36 | If a patient with a stroke is very restless, then ... I regularly must ask  for an order, even though I know what to do. | Restlessnes  s | ... I must obtain an order regularly, even though I  know what to do. | 2,33 | 1,30 | 108 | 2,44 | 1,27 | 64 | 0,08 | 0,607 |
| 102 | U38 | If a patient with a stroke is very restless, then ... cooperation with the  doctors is very well organized. | Restlessnes  s | ... cooperation with the doctors is very well organized. | 2,54 | 1,05 | 108 | 2,51 | 0,98 | 63 | 0,03 | 0,856 |
| 102 | U39 | If a patient with a stroke is very restless, then ... I always know when I  need to call in other people for support. | Restlessnes  s | ... I know when I need to call in other people for  support. | 1,59 | 0,85 | 105 | 1,45 | 0,69 | 64 | 0,17 | 0,253 |
| 102 | U40 | If a patient with a stroke is very restless, then ... optimal care for the  patient is always guaranteed. | Restlessnes  s | ... optimal care of the patient is always guaranteed. | 2,36 | 1,06 | 107 | 2,47 | 0,94 | 64 | 0,10 | 0,505 |
| 102 | U42 | We nursing staff make a very important contribution when dealing with  restless patients in the stroke unit. | Restlessnes  s | Nursing staff make a very important contribution  (from the nursing staff's perspective) | 1,48 | 0,70 | 108 | 1,45 | 0,69 | 64 | 0,04 | 0,796 |
| 102 | U44 | When dealing with restless patients in the stroke unit, the contribution  of nursing is perceived as very important by the doctors. | Restlessnes  s | Contribution of nursing staff is perceived by  doctors (from the nursing staff's perspective) | 2,34 | 1,03 | 107 | 2,35 | 1,05 | 63 | 0,01 | 0,939 |
| 102 | U45 | The procedure for restless patients is first class. | Restlessnes  s | Procedure for symptoms is first class | 2,57 | 1,06 | 108 | 2,61 | 0,94 | 64 | 0,03 | 0,821 |
| 102 | E214 | I can act competently as a nurse when dealing with restless patients. | Restlessnes  s | Nursing staff can act competently (from the nursing  staff's perspective) | 1,93 | 0,86 | 108 | 1,95 | 0,90 | 64 | 0,03 | 0,846 |
| 102 | E35 | If a patient with a stroke has sleep disorders, then ... I know exactly what to do. | Sleep  disorder | ... I know exactly what to do. | 1,96 | 0,92 | 110 | 1,87 | 0,81 | 63 | 0,10 | 0,502 |
| 102 | E36 | If a patient with a stroke has trouble sleeping, then ... I can always react  promptly. | Sleep  disorder | ... I can always react promptly. | 2,06 | 0,90 | 109 | 1,97 | 0,99 | 62 | 0,10 | 0,528 |
| 102 | E37 | If a patient with a stroke has sleep disorders, then ... I can act  independently. | Sleep  disorder | ... I can act independently. | 2,72 | 1,17 | 107 | 2,60 | 1,22 | 62 | 0,10 | 0,524 |
| 102 | E38 | If a patient with a stroke has sleep disorders, then ... I feel competent  in working with the doctors. | Sleep  disorder | ... I feel competent in working with the doctors. | 2,18 | 1,02 | 109 | 2,16 | 1,00 | 61 | 0,02 | 0,904 |
| 102 | E39 | If a patient with a stroke has sleep disorders, then ... I must ask the  doctors regularly for substantive reasons. | Sleep  disorder | ... I must ask the doctors regularly for substantive reasons. | 3,07 | 1,31 | 107 | 2,94 | 1,24 | 63 | 0,11 | 0,493 |
| 102 | E41 | If a patient with a stroke has sleep disorders, then ... I regularly must  ask for an order, even though I know what to do. | Sleep  disorder | ... I must obtain an order regularly, even though I  know what to do. | 2,32 | 1,27 | 109 | 2,35 | 1,22 | 63 | 0,02 | 0,886 |
| 102 | E43 | If a patient with a stroke has sleep disorders, then ... cooperation with  the doctors is very well organized. | Sleep  disorder | ... cooperation with the doctors is very well organized. | 2,41 | 1,08 | 108 | 2,54 | 0,98 | 63 | 0,13 | 0,413 |
| 102 | E44 | If a patient with a stroke has sleep disorders, then ... I know when to  call in other people for support if necessary. | Sleep  disorder | ... I know when to call in other people for support if necessary. | 1,66 | 0,89 | 106 | 1,48 | 0,74 | 63 | 0,22 | 0,149 |
| 102 | E45 | If a patient with a stroke has sleep disorders, then ... optimal care for  the patient is always guaranteed. | Sleep  disorder | ... optimal care for the patient is always guaranteed. | 2,35 | 1,09 | 110 | 2,38 | 0,87 | 63 | 0,03 | 0,861 |
| 102 | E46 | As a nurse, I can act competently in the event of sleep disorders in the  stroke unit. | Sleep  disorder | Nurse can act competently (from the perspective of  the nursing staff) | 2,15 | 0,96 | 109 | 2,05 | 0,85 | 63 | 0,11 | 0,484 |
| 102 | E48 | We nursing staff make a very important contribution to sleep disorders  in the stroke unit. | Sleep  disorder | Nursing staff make a very important contribution  (from the nursing staff's perspective) | 1,72 | 0,88 | 109 | 1,45 | 0,67 | 62 | 0,32 | 0,029 |
| 102 | E50 | In the case of sleep disorders in the stroke unit, the contribution of  nursing care is perceived as important by the doctors. | Sleep  disorder | Contribution of nursing staff is perceived by doctors (from the nursing staff's perspective) | 2,35 | 1,14 | 104 | 2,37 | 0,95 | 59 | 0,02 | 0,872 |
| 102 | E51 | The procedure for sleep disorders is first class. | Sleep  disorder | Procedure for symptom is first class | 2,71 | 1,14 | 108 | 2,57 | 0,90 | 61 | 0,13 | 0,385 |

|  |  | Doctors |  |  | **pre** | | | **post** | | |  |  |
| --- | --- | --- | --- | --- | --- | --- | --- | --- | --- | --- | --- | --- |
| **BG**  **code** | **Item** | **Question text** | **Symptom** | **Cluster** | **M** | **SD** | **N** | **M** | **SD** | **N** | **Cohen's d** | **p- value** |
| 101 | E520 | Note on collaboration with physiotherapists |  |  | 1,54 | 0,69 | 80 | 1,49 | 0,51 | 49 | 0,08 | 0,653 |
| 101 | E521 | Grade for cooperation with medical service |  |  | 1,58 | 0,61 | 78 | 1,62 | 0,82 | 47 | 0,06 | 0,773 |
| 101 | E522 | Grade for cooperation with nursing service |  |  | 2,05 | 0,78 | 79 | 1,92 | 0,74 | 48 | 0,17 | 0,335 |
| 101 | E523 | Grade for cooperation with speech therapists/speech therapists |  |  | 1,48 | 0,73 | 80 | 1,43 | 0,54 | 49 | 0,07 | 0,680 |
| 101 | E524 | Grade for cooperation with occupational therapists |  |  | 1,58 | 0,73 | 80 | 1,53 | 0,54 | 49 | 0,07 | 0,693 |
| 101 | R01 | a. In my area, employees / colleagues are specifically trained. |  |  | 1,84 | 0,60 | 80 | 1,71 | 0,61 | 49 | 0,20 | 0,268 |
| 101 | E503 | e. Employees / colleagues are specifically trained on the topic of  fever in stroke. |  | Targeted training | 1,83 | 0,76 | 80 | 1,42 | 0,58 | 48 | 0,59 | 0,001 |
| 101 | E501 | c. Employees / colleagues are specifically trained on the topic of  blood sugar in stroke. |  | Targeted training | 2,01 | 0,82 | 80 | 1,67 | 0,72 | 48 | 0,44 | 0,014 |
| 101 | E505 | b./g. Employees / colleagues are specifically trained on the topic  of dysphagia in stroke patients. |  | Targeted training | 1,79 | 0,74 | 80 | 1,35 | 0,56 | 48 | 0,64 | 0,000 |
| 101 | E504 | f. Employees / colleagues are specifically trained on the topic of  blood pressure in stroke. |  | Targeted training | 1,35 | 0,58 | 80 | 1,19 | 0,45 | 48 | 0,31 | 0,077 |
| 101 | E500 | b. Employees / colleagues are specifically trained on the topic of  pain in stroke patients. |  | Targeted training | 2,46 | 0,69 | 80 | 2,15 | 0,78 | 47 | 0,43 | 0,025 |
| 101 | E502 | d. Employees / colleagues are specifically trained on the topic of  sleep in stroke patients. |  | Targeted training | 2,64 | 0,58 | 80 | 2,11 | 0,81 | 47 | 0,79 | 0,000 |
| 101 | E506 | h. Employees / colleagues are specifically trained on the topic of  restlessness in the event of a stroke. |  | Targeted training | 2,23 | 0,64 | 80 | 1,73 | 0,68 | 48 | 0,76 | 0,000 |

| 101 | E52 | If a patient with a stroke has an elevated temperature (>37.5°), then  ... I know exactly what to do. | Fever | ... I know exactly what to do. | 1,31 | 0,56 | 81 | 1,29 | 0,50 | 48 | 0,03 | 0,860 |
| --- | --- | --- | --- | --- | --- | --- | --- | --- | --- | --- | --- | --- |
| 101 | E53 | If a patient with a stroke has an elevated temperature (>37.5°), then  ... I can always react promptly. | Fever | ... I can always react promptly. | 1,56 | 0,74 | 81 | 1,40 | 0,68 | 48 | 0,22 | 0,214 |
| 101 | E54 | If a patient with a stroke has an elevated temperature (>37.5°), then  ... I can act independently. | Fever | ... I can act independently. | 1,43 | 0,69 | 80 | 1,25 | 0,48 | 48 | 0,28 | 0,095 |
| 101 | E55 | If a patient with a stroke has an elevated temperature (>37.5°), then  ... I feel competent in working with the nurses. | Fever | ... I feel competent in working with the  nurses. | 1,42 | 0,61 | 81 | 1,35 | 0,53 | 48 | 0,11 | 0,520 |
| 101 | E57 | If a patient with a stroke has an elevated temperature (>37.5°), then  ... I regularly discuss the findings with the nursing staff. | Fever | ... I regularly discuss the findings with  the nursing staff. | 1,56 | 0,65 | 80 | 1,38 | 0,57 | 48 | 0,30 | 0,091 |
| 101 | E59 | If a patient with a stroke has an elevated temperature (>37.5°), then  ... I only receive queries from the nursing staff if this is necessary. | Fever | ... I only receive questions from the  nursing staff if this is necessary. | 1,89 | 0,97 | 81 | 1,58 | 0,77 | 48 | 0,34 | 0,051 |
| 101 | E60 | If a patient with a stroke has an elevated temperature (>37.5°), then  ... cooperation with the nurses is very well organized. | Fever | ... cooperation with the doctors/nursing  staff is very well organized. | 1,70 | 0,82 | 80 | 1,46 | 0,71 | 48 | 0,31 | 0,082 |
| 101 | E61 | If a patient with a stroke has an elevated temperature (>37.5°), then  ... I know when to call in other people for support if necessary. | Fever | ... I know when to call in other people  for support if necessary. | 1,46 | 0,76 | 81 | 1,38 | 0,57 | 48 | 0,12 | 0,489 |
| 101 | E62 | If a patient with a stroke has an elevated temperature (>37.5°), then  ... optimal care for the patient is always guaranteed. | Fever | ... optimal care of the patient is always  guaranteed. | 1,74 | 0,78 | 80 | 1,44 | 0,65 | 48 | 0,41 | 0,021 |
| 101 | E64 | I feel that the nursing staff are competent when it comes to fever in  the stroke unit. | Fever | Nurses can act competently (from the  doctors' point of view) | 1,64 | 0,73 | 81 | 1,35 | 0,56 | 48 | 0,43 | 0,014 |
| 101 | E66 | Nursing staff make a very important contribution to fever management in the stroke unit. | Fever | Nursing staff make a very important contribution (from the doctors' perspective) | 1,41 | 0,61 | 81 | 1,29 | 0,50 | 48 | 0,20 | 0,246 |
| 101 | E68 | The procedure for fever is first class. | Fever | Procedure for symptoms is first class | 1,94 | 0,82 | 79 | 1,73 | 0,79 | 48 | 0,26 | 0,161 |
| 101 | E18 | If a patient with a stroke has elevated blood sugar levels, then ... I  know exactly what to do. | Blood sugar | ... I know exactly what to do. | 1,74 | 0,89 | 81 | 1,65 | 0,81 | 48 | 0,11 | 0,538 |
| 101 | E19 | If a patient with a stroke has elevated blood sugar levels, then ... I  can always react promptly. | Blood sugar | ... I can always react promptly. | 1,77 | 0,87 | 81 | 1,69 | 0,80 | 48 | 0,09 | 0,607 |
| 101 | E20 | If a patient with a stroke has elevated blood sugar levels, then ... I  can act independently. | Blood sugar | ... I can act independently. | 1,79 | 0,97 | 81 | 1,63 | 0,84 | 48 | 0,18 | 0,312 |
| 101 | E21 | If a patient with a stroke has elevated blood sugar levels, then ... I  feel competent in working with the nurses. | Blood sugar | ... I feel competent in working with the nurses. | 1,73 | 0,84 | 81 | 1,53 | 0,69 | 47 | 0,25 | 0,153 |
| 101 | E23 | If a patient with a stroke has elevated blood sugar levels, then ... I  regularly discuss the findings with the nursing staff. | Blood sugar | ... I regularly discuss the findings with the nursing staff. | 1,89 | 0,88 | 81 | 1,60 | 0,79 | 48 | 0,34 | 0,061 |
| 101 | E25 | If a patient with a stroke has elevated blood sugar levels, then ... I  only receive questions from the nursing staff if this is necessary. | Blood sugar | ... I only receive questions from the nursing staff if this is necessary. | 2,16 | 1,02 | 81 | 1,75 | 0,89 | 48 | 0,42 | 0,018 |
| 101 | E26 | If a patient with a stroke has elevated blood sugar levels, then ...  cooperation with the nurses is very well organized. | Blood sugar | ... cooperation with the nurses is very well organized. | 1,80 | 0,83 | 80 | 1,52 | 0,71 | 48 | 0,35 | 0,047 |
| 101 | E27 | If a patient with a stroke has elevated blood glucose levels, then ... I  know when to call in other people for support if necessary. | Blood sugar | ... I know when to call in other people for support if necessary. | 1,60 | 0,75 | 81 | 1,54 | 0,62 | 48 | 0,09 | 0,606 |
| 101 | E28 | If a patient with a stroke has elevated blood glucose levels, then ...  optimal care for the patient is always guaranteed. | Blood sugar | ... optimal care for the patient is always  guaranteed. | 1,95 | 0,79 | 81 | 1,77 | 0,75 | 48 | 0,23 | 0,200 |
| 101 | E30 | I feel that the nursing staff are competent when it comes to  elevated blood sugar levels in the stroke unit. | Blood sugar | Nurses can act competently (from the doctors' point of view) | 1,81 | 0,80 | 80 | 1,54 | 0,65 | 48 | 0,36 | 0,039 |
| 101 | E32 | Nursing staff make a very important contribution to blood glucose management in the stroke unit. | Blood sugar | Nursing staff make a very important contribution (from the doctors' perspective) | 1,44 | 0,63 | 80 | 1,31 | 0,59 | 48 | 0,20 | 0,261 |
| 101 | E34 | The procedure for elevated blood sugar levels is first class. | Blood sugar | Procedure for symptoms is first class | 2,09 | 0,84 | 78 | 1,92 | 0,79 | 48 | 0,21 | 0,248 |
| 101 | E86 | If a patient with dysphagia comes to the stroke unit, then ... I know  exactly what to do regarding dysphagia. | Dysphagia | ... I know exactly what to do. | 2,14 | 1,05 | 79 | 1,90 | 0,90 | 48 | 0,24 | 0,169 |
| 101 | E87 | If a patient with a swallowing disorder/ dysphagia comes to the  stroke unit, then ... I can always react promptly. | Dysphagia | ... I can always react promptly. | 2,20 | 1,03 | 79 | 1,98 | 0,91 | 48 | 0,23 | 0,205 |
| 101 | C01 | If a patient with a swallowing disorder/ dysphagia comes to the  stroke unit, then ... I can act independently. | Dysphagia | ... I can act independently. | 2,48 | 1,10 | 79 | 2,00 | 0,95 | 48 | 0,46 | 0,010 |
| 101 | C02 | If a patient with a swallowing disorder/ dysphagia comes to the stroke unit, then I feel competent in working with the  nurses. | Dysphagia | ... I feel competent in cooperation with the nurses. | 2,12 | 0,90 | 78 | 1,75 | 0,76 | 48 | 0,43 | 0,016 |
| 101 | C03 | If a patient with a swallowing disorder/ dysphagia comes to the  stroke unit, then ... I regularly discuss the findings with the doctors. | Dysphagia | ... I regularly discuss the findings with  the nursing staff. | 2,06 | 0,95 | 79 | 1,73 | 0,74 | 48 | 0,38 | 0,029 |
| 101 | C05 | If a patient with a swallowing disorder/ dysphagia comes to the stroke unit, then I regularly only receive queries from the nursing  staff if this is necessary. | Dysphagia | ... I only receive questions from the nursing staff when this is necessary. | 2,23 | 1,09 | 78 | 1,81 | 0,76 | 48 | 0,43 | 0,013 |
| 101 | C06 | If a patient with dysphagia comes to the stroke unit, then ...  cooperation with the nurses is very well organized | Dysphagia | ... cooperation with the nurses is very well organized. | 2,06 | 1,00 | 78 | 1,71 | 0,74 | 48 | 0,39 | 0,024 |
| 101 | U10 | If a patient with a swallowing disorder/ dysphagia comes to the stroke unit, then ... I feel competent in working with the speech  therapists/speech therapists/doctors. | Dysphagia | ... I feel competent in working with speech therapists/speech therapists/doctors. | 1,81 | 0,95 | 79 | 1,48 | 0,71 | 48 | 0,38 | 0,028 |
| 101 | U11 | If a patient with a swallowing disorder/ dysphagia comes to the stroke unit, then ... I only receive questions from the speech  therapists/speech therapists if this is necessary. | Dysphagia | ... I only receive questions from the speech and language therapists if this is  necessary. | 1,78 | 0,98 | 79 | 1,40 | 0,61 | 48 | 0,45 | 0,007 |
| 101 | U12 | If a patient with a swallowing disorder/ dysphagia comes to the stroke unit, then ... I regularly discuss the findings with the speech  therapists/speech therapists/doctors. | Dysphagia | ... I regularly discuss the findings with the speech therapists/speech therapists/doctors. | 1,63 | 0,88 | 79 | 1,38 | 0,73 | 48 | 0,31 | 0,078 |
| 101 | U13 | If a patient with a swallowing disorder/ dysphagia comes to the stroke unit, then ... cooperation with the speech therapists/speech  therapists/doctors is very well organized | Dysphagia | ... cooperation with the speech therapists/speech therapists/doctors is  very well organized | 1,59 | 0,82 | 79 | 1,35 | 0,64 | 48 | 0,32 | 0,067 |
| 101 | U14 | If a patient with dysphagia comes to the stroke unit, then ... I know  when to call in other people for support if necessary. | Dysphagia | ... I know when I need to call in another  people for support if necessary. | 1,63 | 0,80 | 79 | 1,48 | 0,62 | 48 | 0,21 | 0,229 |
| 101 | U15 | If a patient with a swallowing disorder/ dysphagia is admitted to the stroke unit, then ... optimal care for the patient is always  guaranteed. | Dysphagia | ... optimal care of the patient is always guaranteed. | 1,83 | 0,82 | 77 | 1,69 | 0,69 | 48 | 0,19 | 0,294 |
| 101 | U19 | I feel that the nursing staff are competent in the assessment of  dysphagia in the stroke unit. | Dysphagia | Nurses can act competently (from the  doctors' point of view) | 2,09 | 0,76 | 78 | 1,83 | 0,75 | 48 | 0,34 | 0,067 |
| 101 | U21 | Nursing staff make a very important contribution to the assessment of dysphagia in the stroke unit. | Dysphagia | Nurses make a very important contribution (from the doctors'  perspective) | 1,73 | 0,80 | 79 | 1,54 | 0,65 | 48 | 0,26 | 0,141 |

| 101 | E69 | If a patient with a stroke has high blood pressure, then ... I know  exactly what to do. | Blood  pressure | ... I know exactly what to do. | 1,40 | 0,65 | 81 | 1,29 | 0,50 | 48 | 0,17 | 0,313 |
| --- | --- | --- | --- | --- | --- | --- | --- | --- | --- | --- | --- | --- |
| 101 | E70 | If a patient with a stroke has elevated blood pressure, then ... I can  always react promptly. | Blood  pressure | ... I can always react promptly. | 1,54 | 0,73 | 81 | 1,42 | 0,61 | 48 | 0,18 | 0,293 |
| 101 | E71 | If a patient with a stroke has elevated blood pressure, then ... I can  act independently. | Blood  pressure | ... I can act independently. | 1,40 | 0,59 | 80 | 1,27 | 0,49 | 48 | 0,23 | 0,185 |
| 101 | E72 | If a patient with a stroke has elevated blood pressure, then ... I feel  competent in working with the doctors. | Blood  pressure | ... I feel competent in working with the nurses. | 1,44 | 0,63 | 81 | 1,35 | 0,53 | 48 | 0,15 | 0,384 |
| 101 | E74 | If a patient with a stroke has elevated blood pressure values, then ...  I regularly discuss the findings with the nursing staff. | Blood  pressure | ... I regularly discuss the findings with the nursing staff. | 1,58 | 0,69 | 81 | 1,35 | 0,60 | 48 | 0,34 | 0,053 |
| 101 | E76 | If a patient with a stroke has elevated blood pressure values, then ...  I only receive questions from the nursing staff if this is necessary. | Blood  pressure | ... I only receive questions from the nursing staff if this is necessary. | 1,99 | 0,99 | 81 | 1,71 | 0,87 | 48 | 0,29 | 0,099 |
| 101 | E77 | If a patient with a stroke has elevated blood pressure values, then ...  cooperation with the nurses is very well organized. | Blood  pressure | ... cooperation with the nurses is very well organized. | 1,70 | 0,83 | 81 | 1,42 | 0,65 | 48 | 0,37 | 0,031 |
| 101 | E78 | If a patient with a stroke has high blood pressure, then ... I know  when to call in other people for support if necessary. | Blood  pressure | ... I know when to call in other people for support if necessary. | 1,44 | 0,65 | 80 | 1,35 | 0,53 | 48 | 0,14 | 0,430 |
| 101 | E79 | If a patient with a stroke has elevated blood pressure values, then ...  optimal care for the patient is always guaranteed. | Blood  pressure | ... optimal care for the patient is always  guaranteed. | 1,83 | 0,79 | 81 | 1,58 | 0,79 | 48 | 0,31 | 0,094 |
| 101 | E81 | I feel that the nursing staff are competent in dealing with elevated  blood pressure values in the stroke unit. | Blood  pressure | Nursing staff can act competently (from  the doctors' point of view) | 1,72 | 0,83 | 81 | 1,48 | 0,65 | 48 | 0,31 | 0,074 |
| 101 | E83 | Nursing staff make a very important contribution to blood pressure management in the stroke unit. | Blood pressure | Nursing staff make a very important contribution (from the doctors' point of view) | 1,36 | 0,62 | 81 | 1,31 | 0,62 | 48 | 0,07 | 0,689 |
| 101 | E85 | The procedure for elevated blood pressure values is first class. | Blood  pressure | Procedure for symptoms is first class | 1,82 | 0,78 | 79 | 1,79 | 0,82 | 48 | 0,04 | 0,834 |
| 101 | E01 | If a patient with a stroke has severe pain, then ... I know exactly  what to do. | Pain | ... I know exactly what to do. | 1,90 | 0,73 | 81 | 1,71 | 0,65 | 48 | 0,27 | 0,124 |
| 101 | E02 | If a patient with a stroke has severe pain, then ... I can always react  promptly. | Pain | ... I can always react promptly. | 1,96 | 0,83 | 81 | 1,75 | 0,67 | 48 | 0,28 | 0,113 |
| 101 | E03 | If a patient with a stroke has severe pain, then ... I can act  independently. | Pain | ... I can act independently. | 1,65 | 0,71 | 81 | 1,50 | 0,65 | 48 | 0,22 | 0,212 |
| 101 | E04 | If a patient with a stroke has severe pain, then ... I feel competent in  working with the nurses. | Pain | ... I feel competent in working with the  nurses. | 1,80 | 0,83 | 81 | 1,65 | 0,64 | 48 | 0,21 | 0,230 |
| 101 | E06 | If a patient with a stroke has severe pain, then ... I regularly discuss  the findings with the nursing staff. | Pain | ... I regularly discuss the findings with  the nursing staff. | 1,95 | 0,91 | 81 | 1,77 | 0,90 | 48 | 0,20 | 0,278 |
| 101 | E08 | If a patient with a stroke has severe pain, then ... I only receive  questions from the nursing staff if this is necessary. | Pain | ... I only receive questions from the  nursing staff if this is necessary. | 2,42 | 1,06 | 81 | 2,02 | 1,02 | 48 | 0,38 | 0,037 |
| 101 | E09 | If a patient with a stroke has severe pain, then ... cooperation with  the nurses is very well organized. | Pain | ... cooperation with the nurses  is very well organized. | 2,11 | 0,79 | 81 | 1,81 | 0,76 | 48 | 0,38 | 0,036 |
| 101 | E10 | If a patient with a stroke has severe pain, then ... I know when to call  in other people for support if necessary. | Pain | ... I know when to call in other people  for support if necessary. | 1,68 | 0,76 | 79 | 1,70 | 0,72 | 47 | 0,02 | 0,891 |
| 101 | E11 | If a patient with a stroke has severe pain, then ... optimal care for  the patient is always guaranteed. | Pain | ... optimal care for the patient is always  guaranteed. | 2,30 | 0,86 | 81 | 2,02 | 0,84 | 48 | 0,32 | 0,077 |
| 101 | E13 | I feel that the nursing staff are competent in pain management on  the stroke unit. | Pain | Nurses can act competently (from the  doctors' point of view) | 2,17 | 0,75 | 81 | 2,00 | 0,71 | 48 | 0,23 | 0,196 |
| 101 | E15 | Nursing staff make a very important contribution to pain management in the stroke unit. | Pain | Nursing staff make a very important contribution (from the doctors' perspective) | 1,67 | 0,79 | 81 | 1,48 | 0,68 | 48 | 0,25 | 0,159 |
| 101 | E17 | Our pain management is first class. | Pain | Symptom process is first class | 2,72 | 0,85 | 79 | 2,40 | 0,89 | 48 | 0,38 | 0,045 |
| 101 | U30 | If a patient with a stroke is very restless, then ... I know exactly what  to do. | Restlessness | ... I know exactly what to do. | 2,15 | 0,84 | 80 | 1,85 | 0,71 | 48 | 0,37 | 0,036 |
| 101 | U31 | If a patient with a stroke is very restless, then ... is always responded  to promptly. | Restlessness | ... I can always react promptly. | 2,20 | 0,89 | 80 | 1,90 | 0,72 | 48 | 0,37 | 0,037 |
| 101 | U32 | If a patient with a stroke is very restless, then ... I can react  independently. | Restlessness | ... I can act independently. | 1,86 | 0,92 | 80 | 1,65 | 0,73 | 48 | 0,25 | 0,144 |
| 101 | U33 | If a patient with a stroke is very restless, then ... I feel competent in  working with the nurses. | Restlessness | ... I feel competent in working with the  nurses. | 2,06 | 0,97 | 80 | 1,75 | 0,60 | 48 | 0,37 | 0,026 |
| 101 | U35 | If a patient with a stroke is very restless, then ... I regularly discuss  the findings with the nursing staff. | Restlessness | ... I regularly discuss the findings with  the nursing staff. | 1,89 | 0,76 | 80 | 1,65 | 0,73 | 48 | 0,32 | 0,077 |
| 101 | U37 | If a patient with a stroke is very restless, then ... I only receive  questions from the nursing staff if this is necessary. | Restlessness | ... I only receive questions from the  nursing staff if this is necessary. | 2,51 | 1,21 | 80 | 2,13 | 0,91 | 48 | 0,35 | 0,043 |
| 101 | U38 | If a patient with a stroke is very restless, then ... cooperation with  the nurses is very well organized. | Restlessness | ... cooperation with the nurses  is very well organized. | 2,28 | 1,02 | 80 | 1,81 | 0,64 | 48 | 0,52 | 0,002 |
| 101 | U39 | If a patient with a stroke is very restless, then ... I always know when  I need to call in other people for support. | Restlessness | ... I know when I need to call in another  people for support if necessary. | 1,80 | 0,99 | 80 | 1,81 | 0,76 | 48 | 0,01 | 0,936 |
| 101 | U40 | If a patient with a stroke is very restless, then ... optimal care for the  patient is always guaranteed. | Restlessness | ... optimal care of the patient is always  guaranteed. | 2,53 | 1,00 | 79 | 2,06 | 0,84 | 48 | 0,50 | 0,005 |
| 101 | U41 | I feel that the nursing staff are competent in dealing with restless  patients. | Restlessness | Nursing staff can act competently (from  the doctors' point of view) | 2,43 | 0,99 | 80 | 2,10 | 0,83 | 48 | 0,34 | 0,052 |
| 101 | U43 | Nursing staff make a very important contribution when dealing with restless patients in the stroke unit. | Restlessness | Nursing staff make a very important contribution (from the doctors' perspective) | 1,78 | 0,89 | 80 | 1,58 | 0,68 | 48 | 0,24 | 0,171 |
| 101 | U45 | The procedure for restless patients is first class. | Restlessness | Procedure for symptoms is first class | 2,63 | 0,99 | 76 | 2,33 | 0,83 | 48 | 0,32 | 0,074 |
| 101 | E35 | If a patient with a stroke has sleep disorders, then ... I know exactly  what to do. | Sleep  disorder | ... I know exactly what to do. | 2,27 | 0,99 | 81 | 1,92 | 0,85 | 48 | 0,38 | 0,033 |
| 101 | E36 | If a patient with a stroke has trouble sleeping, then ... I can always  react promptly. | Sleep  disorder | ... I can always react promptly. | 2,33 | 0,97 | 81 | 2,04 | 0,85 | 48 | 0,31 | 0,077 |
| 101 | E37 | If a patient with a stroke has difficulty sleeping, then ... I can act  independently. | Sleep  disorder | ... I can act independently. | 1,91 | 0,96 | 81 | 1,60 | 0,61 | 48 | 0,36 | 0,027 |
| 101 | E38 | If a patient with a stroke has sleep disorders, then ... I feel  competent in working with the nurses. | Sleep  disorder | ... I feel competent in working with the  nurses. | 2,14 | 0,93 | 81 | 1,68 | 0,59 | 47 | 0,55 | 0,001 |
| 101 | E40 | If a patient with a stroke has sleep disorders, then ... I regularly  discuss the findings with the nursing staff. | Sleep  disorder | ... I regularly discuss the findings with  the nursing staff. | 2,18 | 0,95 | 80 | 1,77 | 0,76 | 47 | 0,46 | 0,009 |
| 101 | E42 | If a patient with a stroke has sleep disorders, then ... I only receive  questions from the nursing staff if this is necessary. | Sleep  disorder | ... I only receive questions from the  nursing staff if this is necessary. | 2,38 | 1,10 | 81 | 2,04 | 0,93 | 47 | 0,33 | 0,066 |
